# USP49 deubiquitinase regulates the mitotic spindle checkpoint and prevents aneuploidy

**DOI:** 10.1038/s41419-023-05600-x

**Published:** 2023-01-26

**Authors:** Diana Campos-Iglesias, Julia M. Fraile, Gabriel Bretones, Alejandro A. Montero, Elena Bonzon-Kulichenko, Jesús Vázquez, Carlos López-Otín, José M. P. Freije

**Affiliations:** 1 Departamento de Bioquímica y Biología Molecular, Instituto Universitario de Oncología del Principado de Asturias (IUOPA), Universidad de Oviedo, Oviedo, Spain; 2 Centro de Investigación Biomédica en Red de Cáncer (CIBERONC), Madrid, Spain; 3 Instituto de Investigación Sanitaria del Principado de Asturias (ISPA), Oviedo, Spain; 4 Elasmogen Ltd, Liberty Building, Foresterhill Road, Aberdeen, AB25 2ZP UK; 5 Biochemistry Section, Regional Center for Biomedical Research (CRIB), Faculty of Environmental Sciences and Biochemistry, University of Castilla-La Mancha, Avda. Carlos III s/n, 45071 Toledo, Spain; 6 Laboratorio de Proteómica Cardiovascular, Centro Nacional de Investigaciones Cardiovasculares (CNIC), Madrid, Spain; 7 Centro de Investigación Biomédica en Red de Enfermedades Cardiovasculares (CIBERCV), Madrid, Spain

**Keywords:** Deubiquitylating enzymes, Mitosis

## Abstract

The spindle assembly checkpoint (SAC) is an essential mechanism that ensures the accurate chromosome segregation during mitosis, thus preventing genomic instability. Deubiquitinases have emerged as key regulators of the SAC, mainly by determining the fate of proteins during cell cycle progression. Here, we identify USP49 deubiquitinase as a novel regulator of the spindle checkpoint. We show that loss of USP49 in different cancer cell lines impairs proliferation and increases aneuploidy. In addition, USP49-depleted cells overcome the arrest induced by the SAC in the presence of nocodazole. Finally, we report new binding partners of USP49, including ribophorin 1, USP44, and different centrins.

## Introduction

Ubiquitination is a dynamic and reversible process coordinated by the action of ubiquitinating and deubiquitinating enzymes (DUBs) that add or remove ubiquitin moieties, respectively. The human genome encodes 105 proteins classified as DUBs, and among them, the USPs comprise the largest and most diverse family, with 56 members [1]. Due to their wide functional diversity, USPs have critical influence on the regulation of multiple biological processes which are often dysregulated in human malignancies, such as chromatin remodeling, DNA damage repair, RNA maturation and protein synthesis, signal transduction, and cell cycle progression [2]. In this regard, the ubiquitously expressed USP49 deubiquitinase has been related to AKT signaling and DNA damage response regulation, through its interaction with FKBP51 and p53, respectively [3, 4]. Additionally, USP49 has also been implicated in H2B ubiquitination process [5], as well as in the cellular responses to HSV-1 and HIV-1 infection [6, 7]. Moreover, it has been recently proposed that USP49 is transcriptionally activated by c-Myc in colorectal cancer cells [8].

The presence of an abnormal number of chromosomes within the cell, known as aneuploidy, is frequently found in human cancer. In this context, the spindle assembly checkpoint (SAC) plays an essential role, as it ensures the accurate chromosome segregation during mitosis, therefore preventing aneuploidy [9]. During mitosis, the E3 ubiquitin ligase APC/C (anaphase-promoting complex), together with its coactivator CDC20 (also known as CDH1), controls the order of events that ensures accurate chromosome segregation [10]. When bound to Cdc20, the APC^Cdc20^ complex promotes cells to enter anaphase by inducing the degradation of securin and cyclin B, a regulatory subunit of the mitotic-cyclin dependent kinase 1 (Cdk1) [11]. In this sense, several USPs have direct or indirect roles in mitotic progression and checkpoint regulation, carrying out their functions through different key mitotic regulatory proteins [12].

In this work, we provide evidence regarding the crucial role of USP49 deubiquitinase in cell cycle regulation and, consequently, in cellular homeostasis maintenance. Specifically, we show that the depletion of USP49 in different cancer cell lines impairs proliferation. In addition, USP49-depleted cells exhibit an increased number of chromosome segregation errors, and show an impaired arrest in the presence of nocodazole. Moreover, we describe new binding partners of this deubiquitinase, including ribophorin 1, USP44, and different centrins. Taken together, these data suggest a novel role for USP49 in the regulation of the mitotic spindle checkpoint function.

## Materials and methods

### Cell culture

HEK-293T, HeLa, HCT116, U2OS, NCI-H661 and MDA-MB-231 cell lines were purchased from the American Type Culture Collection. Cells were routinely maintained in Dulbecco’s modified Eagle’s medium containing 10% fetal bovine serum (FBS), 1% penicillin-streptomycin-L-glutamine and 1% antibiotic-antimycotic (Life Technologies).

### Plasmids and lentiviral infection

USP49Long and USP49Short cDNAs were cloned into pEGFP and pCDH-GFP empty vectors. For shRNA-resistant *USP49* cloning, silent mutations were introduced into sh*USP49*.79 target sequence using Q5® Site-Directed Mutagenesis Kit (New England Biolabs) with the following primers: 5ʹ-AACCTGCATCTCATGCAATTACAAATCCAATAC-3ʹ and 5ʹ-ACTTGGCTTAGCAGCTGCCCATGAAATATG-3ʹ. The USP49 mutant constructs (W158A and C262A) were generated by using QuikChange II Site-Directed Mutagenesis Kit (Agilent) with the following primers: USP49W158A 5ʹ-GCCAGGACGCTGCGGCTGGCGTTCGAGAAGAGCTCCCGGGG-3ʹ and 5ʹ-CCCCGGGAGCTCTTCTCGAACGCCAGCCGCAGCGTCCTGGC-3ʹ; USP49C262A 5ʹ-GCGCAACCTGGGCAACACCGCCTACATGAACTCCATCCTCC-3ʹ and 5ʹ-GGAGGATGGAGTTCATGTAGGCGGTGTTGCCCAGGTTGCGC-3ʹ. To perform immunoprecipitation experiments, pcDNA3.1 plasmids containing RPN1 and CETN3 cDNAs fused with FLAG-Tag were purchased from GeneScript. USP44-FLAG-HA, CETN1 and CETN2 plasmids were provided by Addgene. The sequences of *USP49*-specific shRNAs were as follows: sh*USP49*.76 5ʹ-CTAGAGGGAAATTACATACATCTCGAGATGTATGTAATTTCCCTCTAG-3ʹ; sh*USP49*.77 5ʹ-CTAGAGGGAAATTACATACATCTCGAGATGTATGTAATTTCCCTCTAG-3ʹ; sh*USP49*.79 5ʹ-CTCAGTCAGGTCACATGTATACTCGAGTATACAGTGACCTGACTGAG-3ʹ. GFP-Tubulin plasmid was purchased from Addgene.

For lentiviral infection, HEK-293T cells were transfected with the corresponding vectors along with the second-generation packaging plasmid psPAX2 and the VSV-G envelope expressing plasmid pMD2.G (Addgene) using Lipofectamine and Plus reagents (Invitrogen). Cell supernatants were collected at 24 and 48 h and added to previously seeded cells, supplemented with 0.8 µg/ml polybrene (Millipore). Stably transduced cells were selected with puromycin at a final concentration of 1 µg/ml.

### Cell proliferation assay

To quantify cell proliferation, 5000 (in the case of HEK293T and HeLa), 2500 (in the case of U2OS, NCI-H661, and MDA-MB-231), or 2000 (in the case of HCT116) cells per well were seeded into 96-well plates, and incubated at 37 ^o^C, 5% CO_2_ for 5 or 10 consecutive days. Then, at the desired time points, a Cell Titer 96 Non-Radioactive cell proliferation kit was used following the manufacturer’s instructions (Promega).

### Cell cycle analysis

Cells were harvested by trypsinization, washed with cold PBS 1× and resuspended in 0.5 ml 70% cold-ethanol that was incorporated dropwise. After 2 h of incubation at 4 ^o^C, cells were spinned down and washed twice with PBS + 1% FBS. Then, 1 ml of 200 µg/ml RNAse and 50 µg/ml propidium iodide mix in PBS was added, followed by 30 min of incubation at 37 ^o^C protected from light. Flow cytometry was performed using a Cytomics FC500 (Beckman Coulter) and data were analyzed using ModFit software (Verity Software House Inc.). To quantify the mitotic progression upon nocodazole block, cells were harvested and processed 16 h after treatment with 100 ng/ml nocodazole (Sigma).

### Chromosome spreads

HCT116 cells were treated with 0.1 µg/ml colcemid (Roche) for 60 min, harvested, treated with hypotonic 0.56% KCl solution for 30 min at 37 ^o^C, and fixed in methanol:acetic acid solution (3:1). Then, preparations were centrifuged 5 min at 100 × *g*, decanted, and washed with 6 ml of methanol:acetic acid solution (3:1). After 30 min of incubation on ice, two more washing steps were performed. Spreads were prepared by dropping cells from ∼30 cm onto glass slides, which were pre-heated over boiling water, allowed to dry, and mounted in DAPI mixed with Prolong Gold Antifade mountant.

### Immunoprecipitation and proteomic studies

To perform RPN1 immunoprecipitation, HEK-293T cells were transfected with pEGFP, pEGFP-USP49Long, or pEGFP-USP49Short plasmids together with RPN1-FLAG. For USP44 and centrins immunoprecipitation, cells were transfected with pCDH-GFP, pCDH-GFP-USP49Long, or pCDH-GFP-USP49Short plasmids together with CETN1-FLAG, CETN2-FLAG, CETN3-FLAG or USP44-FLAG. 24 h after transfection, cells were lysed with co-IP buffer (150 mM NaCl, 20 mM Tris-HCl pH 7.4, 1% NP-40, 1 mM MgCl_2_) supplemented with Benzonase® (Sigma), and protease and phosphatase inhibitors. Then, protein extracts were precleared for 4 h at 4 ^o^C with Dynabeads^TM^ Protein G (Life Technologies), and incubated in rotation with mouse anti-FLAG (F1804, Sigma), control IgG (sc-2025, Santa Cruz) or rabbit anti-CETN3 (HPA035608, Atlas Antibodies) antibodies overnight at 4 ^o^C. Beads were next washed several times with co-IP buffer using a magnet DynaMag^TM^ (Invitrogen), added to protein extracts, and incubated in rotation 4 h at RT. Then, the beads-antibody-antigen complex was washed six times with co-IP buffer and boiled for 10 min in 2x SDS loading buffer. For proteomic analysis, HEK-293T were transfected with pEGFP-USP49Long, pEGFP-USP49Short, or pEGFP empty plasmids. Cells were lysed with co-IP buffer, and protein extracts were precleared for 2 h and incubated with anti-GFP-conjugated Dynabeads for 1 h at 4 ^o^C (anti-GFP from Clontech, 632592). Beads were then washed, and bound proteins were released by boiling in 2× Laemmli buffer. Immunoprecipitates were subjected to protein digestion followed by nano-liquid chromatography, coupled to mass spectrometry for protein identification and quantification by peptide counting as previously described [13].

### Western blot analysis

Western Blot analyses were performed using the following antibodies: anti-Cyclin B1 (4138P, Cell Signaling), anti-β-actin (A5441, Sigma), anti-Flag (2368, Cell Signaling), anti-USP49 (HPA030255, Atlas Antibodies), anti-CETN3 (HPA035608, Atlas Antibodies), IRDye 680RD goat anti-mouse (926–68070, Li-COR), IRDye 680RD goat anti-rabbit (926–68071, Li-COR), and IRDye 800CW goat anti-rabbit (926–32211, Li-COR).

### Immunofluorescence and live-cell microscopy

A construct encoding GFP-tagged H2B (Addgene) was stably transfected into HCT116 and U2OS cells, and cells were then transduced with sh*USP49*.77, sh*USP49*.79, or pLKO.1 vectors. For analyzing mitotic abnormalities, U2OS cells were fixed with 4% formaldehyde in PBS 1x for 10 min and permeabilized with PBS 1x containing 0.5% Triton X-100 and 5% FBS for 15 min. Anti-pericentrin (1:100, ab4448, Abcam) and goat anti-rabbit IgG Alexa Fluor 647 (1:500, A21245, Invitrogen) antibodies were used to mark centrosomes shown in Fig. 2E. Cells were washed with PBST and counterstained with DAPI (Roche, 10236276001, 1 μg/ml in PBS). Images were taken using 40× magnification on a Zeiss AxioObserver microscope. For measuring the time from NEB to anaphase onset, HCT116 cells were synchronized with double thymidine (Sigma) block (2 nM thymidine for 18 h, release for 9 h, 2 mM thymidine for 17 h, release for 6 h). The time from NEB to anaphase onset was measured by time-lapse microscopy using Axioplan-2 fluorescence microscope (Zeiss).

### Statistical analysis

Results are represented as mean ± SEM. Data were analyzed for normal distribution using Saphiro-Wilk test. Differences between two groups were compared by parametric two-tailed Student’s *t* test or non-parametric Mann–Whitney test. Multiple comparison one-way ANOVA test was used when performing USP49 proliferation experiments. Statistical tests were performed using GraphPad Prism software. *p < 0.05; **p < 0.01; ***p < 0.001; ****p < 0.0001.

## Results and discussion

### USP49 loss impairs cell proliferation and leads to aneuploidy

**Fig. 1:**
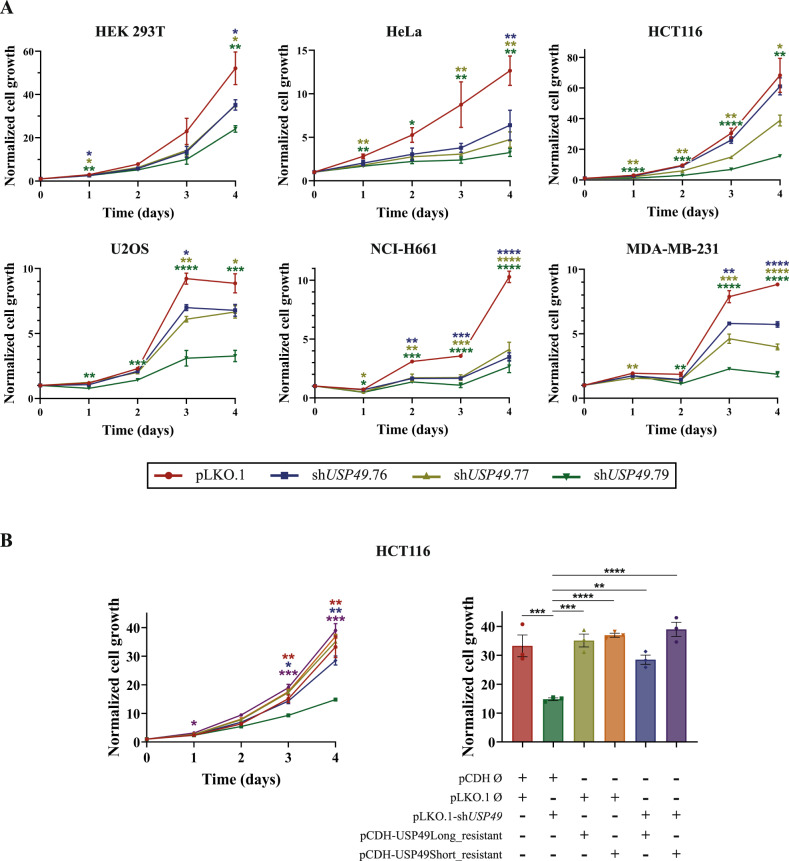
USP49 depletion impairs cell proliferation. **A** MTT proliferation assay in HEK-293T (upper left), HeLa (upper middle), HCT116 (upper right), U2OS (lower left), NCI-H661 (lower middle), and MDA-MB-231 (lower right) cell lines transduced with control (pLKO.1) or three *USP49*-specific shRNAs (sh*USP49*.76, sh*USP49*.77, and sh*USP49*.79). Data are presented as mean ± SEM and statistical significance was assessed by using one-way ANOVA test (pLKO.1 set as the control column). All data were obtained from 3 independent experiments. **B** *USP49* shRNA effects on HCT116 proliferation were rescued by the overexpression of both USP49Long and USP49Short resistant cDNAs. Normalized cell growth at day 4 upon overexpression is presented in the bar graph. Data are presented as mean ± SEM and statistical significance was assessed by using one-way ANOVA test (pCDH/sh*USP49* set as the control column). All data were obtained from 3 independent experiments.

**Fig. 2:**
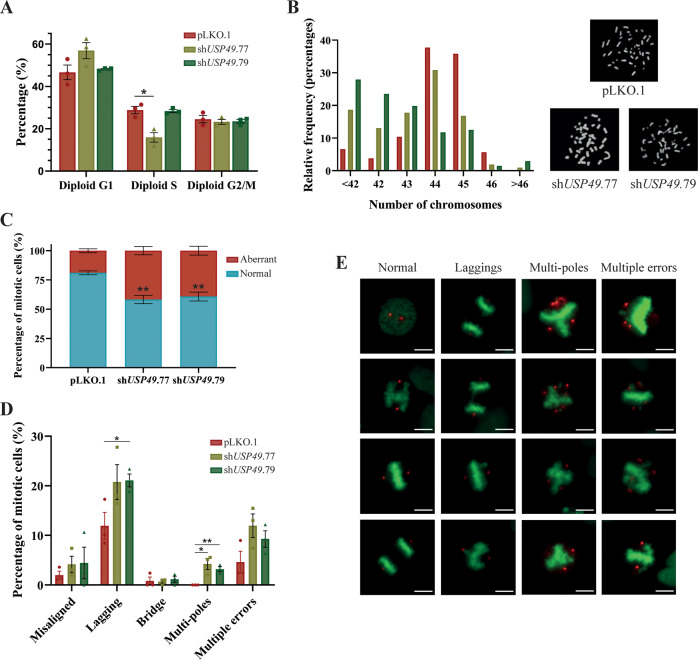
USP49 loss leads to chromosome mis-segregation. **A** HCT116 cells transduced with control or *USP49*-specific shRNAs were harvested for cell cycle analysis by flow cytometry in basal conditions. All data were obtained from 3 independent experiments. Statistical significance was assessed by using two-tailed Student’s *t* test. **B** Chromosome spreads were performed on HCT116 cells transduced with pLKO.1 (*n* = 106), sh*USP49*.77 (*n* = 107), and sh*USP49*.79 (*n* = 136) vectors. Cells were divided into 7 groups according to their chromosome numbers and the relative frequency distribution of each was calculated and represented in a histogram. Chromosome numbers were compared between control and *USP49*-silenced groups using Mann–Whitney test (*p* value <0.001 in pLKO vs. sh*USP49*.77; *p* value <0.001 in pLKO.1 vs sh*USP49*.79). **C**, **D** Quantification of mitotic defects from control or *USP49*-silenced U2OS cells transduced with H2B-GFP. Values represent the mean ± SEM of 3 independent experiments (*n* = 253 pLKO.1; *n* = 251 sh*USP49*.77; *n* = 211 sh*USP49*.79). **E** Representative images of either control or *USP49*-silenced U2OS cells exhibiting normal mitotic progression and the different abnormalities included in C and D. H2G-GFP cells were stained for pericentrin (red). Scale bar = 20 μm.

To investigate the role of USP49 in cellular homeostasis, we stably transduced HEK-293T, HeLa, HCT116, U2OS, NCI-H661, and MDA-MB-231 cancer cells with control or *USP49*-specific shRNAs. As shown in Fig. 1A, USP49 depletion with three independent shRNAs significantly decreased the proliferation rate of all cell lines, an effect which was maintained more than 6 days (Supplementary Fig. S1). In order to rule out undesired off-target effects of the shRNAs, we developed lentiviral vectors containing shRNA-resistant cDNAs encoding two different USP49 isoforms, USP49Long (full-length) and USP49Short (which lacked the Asn residue participating in the catalytic triad) (Supplementary Fig. S2). Back-complementation of both resistant cDNAs in HCT116 cells almost completely restored the phenotype, confirming the specificity of the *USP49* shRNA knock-down (Fig. 1B). Moreover, western blot analysis revealed that ectopically expressed USP49 significantly decreased upon shRNA expression, especially when using sh*USP49*.77 and sh*USP49*.79 (Supplementary Fig. S3). As sh*USP49*.76 presented lower efficiency, we used sh*USP49*.77 and sh*USP49*.79 for most of the subsequent experiments. Furthermore, we have observed that USP49 is located mainly in the nucleus, although it can be also detected in cytoplasm, as it was previously described [7] (Supplementary Fig. S4). Considering the relevance of different USPs in controlling cell cycle progression, we next evaluated whether USP49 depletion influenced mitosis. With this purpose, we infected HCT116 cells with three different shRNAs against USP49 and then analyzed their DNA content by flow cytometry. We did not observe any significant variation in the cell cycle progression of *USP49*-silenced asynchronous populations compared with control cells (Fig. 2A, Supplementary Fig. S5A). However, we found that USP49 downregulation caused a significant increase in aneuploidy (Fig. 2B), suggesting that passage through mitosis in the absence of USP49 would lead to chromosome mis-segregation. In this sense, we observed that USP49 depleted cells showed a higher frequency of mitotic abnormalities, such as lagging chromosomes and multi-polar spindles (Fig. 2C, D, E), confirming the need of USP49 for proper mitotic progression.

### USP49 is required for the mitotic spindle checkpoint

**Fig. 3:**
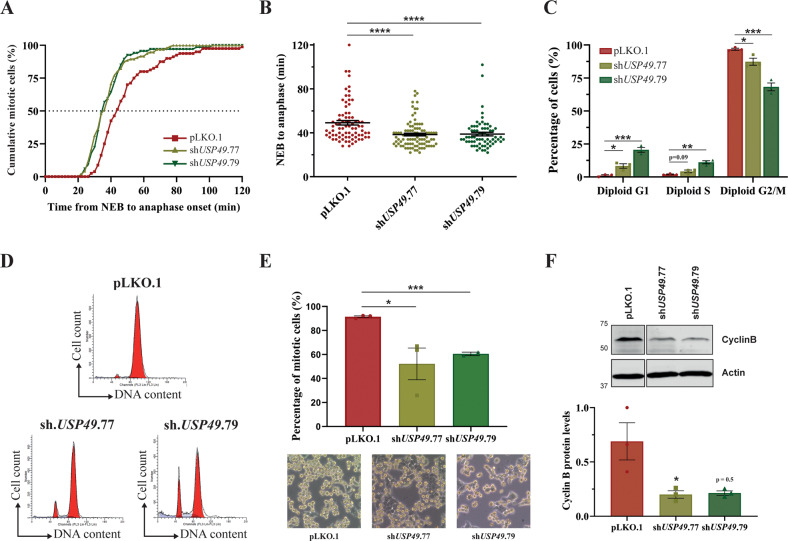
USP49 is required for the spindle checkpoint and its depletion causes mitotic slippage upon nocodazole treatment. **A**, **B** sh*USP49*.77, sh*USP49*.79 or pLKO.1 (control) vectors were transfected into H2B-GFP expressing HCT116 cells. The time from NEB to anaphase onset was measured by time lapse microscopy. The graph in (**A**) represents the cumulative incidence of anaphases at each time point (pLKO.1, *n* = 80; sh*USP49*.77, *n* = 96; sh*USP49*.79, *n* = 67). The time of NEB to anaphase is shown in (**B**). Each point in the scatter plot represents a single cell. Statistical significance was assessed by using Mann–Whitney test. **C** HCT116 cells transduced with control or *USP49*-specific shRNAs were harvested for cell cycle analysis by flow cytometry after 16 h of nocodazole treatment. All data were obtained from 3 independent experiments. Representative examples of the histograms obtained for each condition are shown in (**D**). **E** Percentage of control and USP49-deficient HCT116 cells with rounded morphology upon nocodazole treatment. Five pictures were taken for each condition. Represented data are the mean value ± SEM of three independent experiments (pLKO.1 and sh*USP49*.77), and two replicates (sh*USP49*.79). **F** Western Blot analysis and densitometry quantification of Cyclin B1 protein levels in HCT116 control or *USP49*-silenced cells upon nocodazole treatment. Represented data are the mean value ± SEM of 3 independent experiments. Statistical significance was assessed using two-tailed Student’s *t* test in all cases.

The accurate segregation of sister chromatids is achieved by their linkage to opposite spindle poles prior to cytokinesis, a process which is exhaustively monitored by the SAC, also known as mitotic checkpoint [14]. Thus, in the presence of incorrect or weakly attached kinetochores, this mechanism delays the onset of anaphase, giving cells time to resolve the problematic attachment. Since USP49 depletion clearly affected mitosis but did not seem to induce cell cycle arrest, we next explored the chromosome dynamics and timing of mitosis exit by time-lapse microscopy. After thymidine block synchronization release, H2B-GFP-expressing cells were monitored for 7 h. We observed a significant mitotic acceleration in *USP49* knockdown cells, which exited from mitosis much earlier than control cells (Fig. 3A). Specifically, the mean time between nuclear envelope breakdown (NEB) and anaphase onset upon USP49 depletion was 37 min, compared with 51 min in control cells (Fig. 3B). This shortening in mitotic time suggests that the mitotic checkpoint could be weakened [15]. Taking this into consideration, we next investigated whether *USP49* silencing affected cellular responses to the triggering of the mitotic spindle checkpoint. With this purpose, we analyzed control and *USP49*-knockdown populations after treatment with nocodazole, a microtubule-destabilizing agent, which leads to cellular arrest in prometaphase induced by the SAC, thereby promoting a mitotic checkpoint response [16]. As shown in Figs. 3C, D, 96.85 ± 0.76% of control cells treated with nocodazole displayed a 4 N DNA content (G2/M transition arrest), while 1.17 ± 0.58% of the population remained in a 2 N DNA content (G1 phase). In contrast, *USP49*-knockdown cells did not show that pronounced arrest in G2/M transition (87.35 ± 2.67% for sh*USP49*.77; and 68.29 ± 2.99% for sh*USP49*.79 cells), overriding the mitotic spindle checkpoint triggered by nocodazole (see also Supplementary Fig. S5B for sh*USP49.*76 data). Accordingly, visualization of cell populations by microscopy revealed that approximately 90% of control cells had a mitotic-like rounded shape, whereas only around 60% of USP49-depleted cells exhibited this morphology (Fig. 3E), supporting the previous results. Furthermore, western blot analysis revealed that *USP49* silencing in nocodazole-treated cells led to a decrease in the M-phase-related cyclin (Cyclin B1) expression (Fig. 3F, Supplementary Fig. S5C, Supplementary Fig. S9), which is consistent with an impaired arrest in mitosis in response to nocodazole challenge. From a mechanistic point of view, this mitotic slippage has been proposed to be caused by the rapid degradation of cyclin B during mitotic arrest [17]. However, its upstream regulation is not well understood. Remarkably, mitotic slippage is a major factor in limiting the effectiveness of antimicrotubule chemotherapy drugs that arrest cells in mitosis [18]. Thus, understanding the molecular mechanisms underlying this process could be extraordinarily valuable.

Our results revealed that USP49-depleted cells underwent premature entry into anaphase in unperturbed cell cycles and were less able to arrest in the presence of nocodazole, showing an increased degradation of cyclin B protein levels. Considering that mitotic slippage involves cells exiting mitosis without proper chromosome segregation, it seems feasible that USP49 downregulation effects on chromosome segregation and cell proliferation are a consequence of an inefficient mitotic checkpoint signaling. Thus, we provide evidence that USP49 is essential for spindle checkpoint activation.

### Identification of putative USP49 interacting proteins

**Fig. 4:**
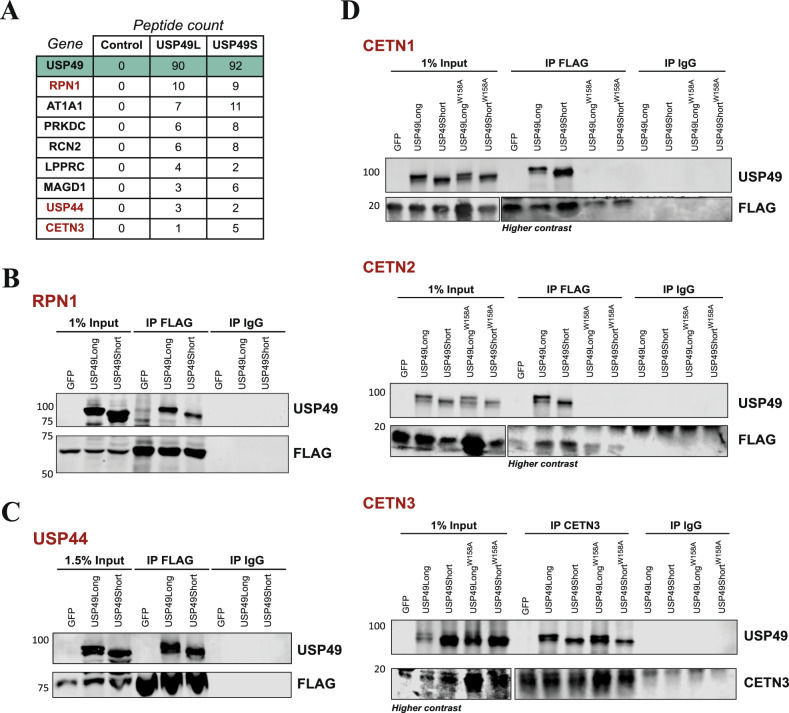
Identification of USP49 interacting proteins. **A** List of top USP49-associated proteins identified by mass spectrometry analysis. HEK-293T cells were co-transfected with GFP, GFP-USP49Long, or GFP-USP49Short in combination with RPN1-FLAG (**B**) or USP44-FLAG (**C**). Cell lysates were then subjected to immunoprecipitation with control IgG or anti-FLAG antibodies. **D** HEK-293T transduced with USP49Long^WT^, USP49Short^WT^, USP49Long^W158A^, and USP49Short^W158A^, together with CETN1 (upper panel), CETN2 (middle panel) or CETN3 (lower panel) were subjected to immunoprecipitation and immunoblotted using the indicated antibodies.

Interestingly, several works have demonstrated that other USPs involved in mitosis control and checkpoint regulation carry out their functions by acting upon different key mitotic regulator proteins. In this sense, USP35 has been reported to regulate mitotic progression through the maintenance of the steady-state levels of Aurora B, a kinase involved in all stages of the mitotic process [19]. Furthermore, USP37 and USP11 seem to influence spindle formation and chromatid resolution by interacting with WAPL and RAE, respectively [20, 21]. Finally, the deubiquitinase USP44 was identified as a key regulator of centrosome separation and chromosome segregation, both dependent on its direct binding to the centriole protein centrin [22]. On this basis, we decided to explore USP49 putative protein interactions within the cell. With this purpose, we used protein extracts from HEK-293T cells transduced with pEGFP, pEGFP-USP49Long and pEGFP-USP49Short vectors to perform GFP immunoprecipitation followed by MS analysis. The complete interaction data is provided in Supplementary Table S1, and the top USP49 putative interactors identified are shown in Fig. 4A. To complement the obtained proteomic data, we searched for USP49 putative protein interactions through the APID database [23]. This analysis revealed a total number of 65 candidates for USP49 interaction (Supplementary Table S2), being some of them also present in our proteomic data. This was the case of RPN1, UBR5, CETN3, USP44, PRS8, LPPRC, PPM1G and IPO7 proteins. To validate the proteomic results, we carried out immunoprecipitation assays of different putative interactor proteins selected among all the obtained data. In this sense, we ectopically expressed GFP, GFP-USP49Long, and GFP-USP49Short isoforms, together with the cDNAs encoding the putative interacting proteins in HEK-293T cells. First, we selected the endoplasmic reticulum protein ribophorin 1 (RPN1), as it was one of the top interacting candidates. As shown in Fig. 4B and Supplementary Fig. S10, USP49 co-immunoprecipitated with RPN1-FLAG, supporting the interaction between both proteins.

Noticeably, the three centrin proteins existing in humans emerged as putative interactors of USP49. These proteins are key components of the centrosomes and mitotic spindle poles, thus having an essential role in cell cycle control and progression. Considering the previous results of USP49 as a regulator of mitosis, we decided to explore whether USP49 interacted with USP44 and centrins. We observed that both USP49 isoforms were specifically recovered in USP44, CETN1, CETN2, and CETN3 immunoprecipitates (Fig. 4C and D, Supplementary Fig. S10), supporting the interaction. Interestingly, the gene coding for USP44, which is the closest paralog of *USP49* (Supplementary Fig. S6 and Supplementary Table S3), has also been related to mitotic abnormalities and interacts directly with centrin 2 [22]. Moreover, the presence of two centrin binding domains (CBDs) in the sequence of USP44 protein reinforces its centrin-binding capacity. Taking this into account, we scanned the sequence of USP49 and found a putative centrin binding domain, which is highly conserved in vertebrates (Supplementary Fig. S7). To assess the centrin-binding capacity of this domain, we developed USP49 mutants (USP49Long^W158A^ and USP49Short^W158A^) and checked their ability to co-precipitate with centrins. We found that both USP49 mutant isoforms were unable to co-precipitate with CETN1 and CETN2 (Fig. 4D, upper and middle panel), whereas there was not any apparent reduction in binding of USP49 mutants to CETN3 (Fig. 4D, lower panel). We hypothesize that W158 is needed for CETN1 and CETN2 binding, whereas binding to CETN3 may involve other residues. Furthermore, we observe that both wild-type USP49Long and catalytic mutant USP49 (USP49Long^C262A^) were specifically co-immunoprecipitated with all three centrins (Supplementary Fig. S8). Overall, these results suggest that this protease is related with centrosomes, possibly impinging on their function and, consequently, on chromosome segregation. However, as reported for USP44, both mitotic checkpoint regulation and chromosome segregation can be regulated independently [24]. Accordingly, further studies will be needed to better understand how USP49-USP44-CETN interaction affects mitotic progression.

In summary, our findings show that USP49 regulates the mitotic spindle checkpoint and prevents aneuploidy. Moreover, we have identified RPN1, USP44, CETN1, CETN2 and CETN3 as new USP49 binding partners, hereby possibly mediating its functions within the cell.

## Supplementary information

Supplementary Figures

Original Data File

Supplementary Tables

Checklist

## Data Availability

The experimental datasets generated and analyzed during this study are included in this published article and its Supplementary Information. Additional data are available from the corresponding author upon request.
