# Supplementary material for: USP49 deubiquitinase regulates the mitotic spindle checkpoint and prevents aneuploidy
Source: Cell Death Dis. 2023 Jan 26;14(1):60. doi: 10.1038/s41419-023-05600-x (PMC9879932; doi:10.1038/s41419-023-05600-x)
Supplement: Supplementary file 1 — Supplementary Figures [file 41419_2023_5600_MOESM1_ESM.pdf]

Supplementary Figures for

USP49 deubiquitinase regulates the mitotic spindle checkpoint and prevents aneuploidy

Diana Campos-Iglesias[1, 2, 3], Julia M. Fraile[4], Gabriel Bretones[1], Alejandro A. Montero[1], Elena Bonzon-Kulichenko[5], Jesús Vázquez[6, 7], Carlos López-Otín[1, 2, 3] and José M. P. Freije[1, 2, 3]

[1]Departamento de Bioquímica y Biología Molecular, Instituto Universitario de Oncología del Principado de Asturias (IUOPA), Universidad de Oviedo, Oviedo, Spain.

[2]Centro de Investigación Biomédica en Red de Cáncer (CIBERONC), Madrid, Spain.

[3] Instituto de Investigación Sanitaria del Principado de Asturias (ISPA), Oviedo, Spain.

[4]Elasmogen Ltd, Liberty Building, Foresterhill Road, Aberdeen AB25 2ZP, U.K.

[5]Biochemistry Section, Regional Center for Biomedical Research (CRIB), Faculty of Environmental Sciences and Biochemistry, University of Castilla-La Mancha, Avda. Carlos III s/n, 45071 Toledo, Spain.

[6]Laboratorio de Proteómica Cardiovascular, Centro Nacional de Investigaciones Cardiovasculares (CNIC), Madrid, Spain.

[7]Centro de Investigación Biomédica en Red de Enfermedades Cardiovasculares (CIBERCV), Madrid, Spain.

Correspondence to:

José M.P. Freije (jmpf@uniovi.es) or Carlos López-Otín (clo@uniovi.es)

Departamento de Bioquímica y Biología Molecular

Universidad de Oviedo, 33006-Oviedo, Spain

Supplementary Figure S1

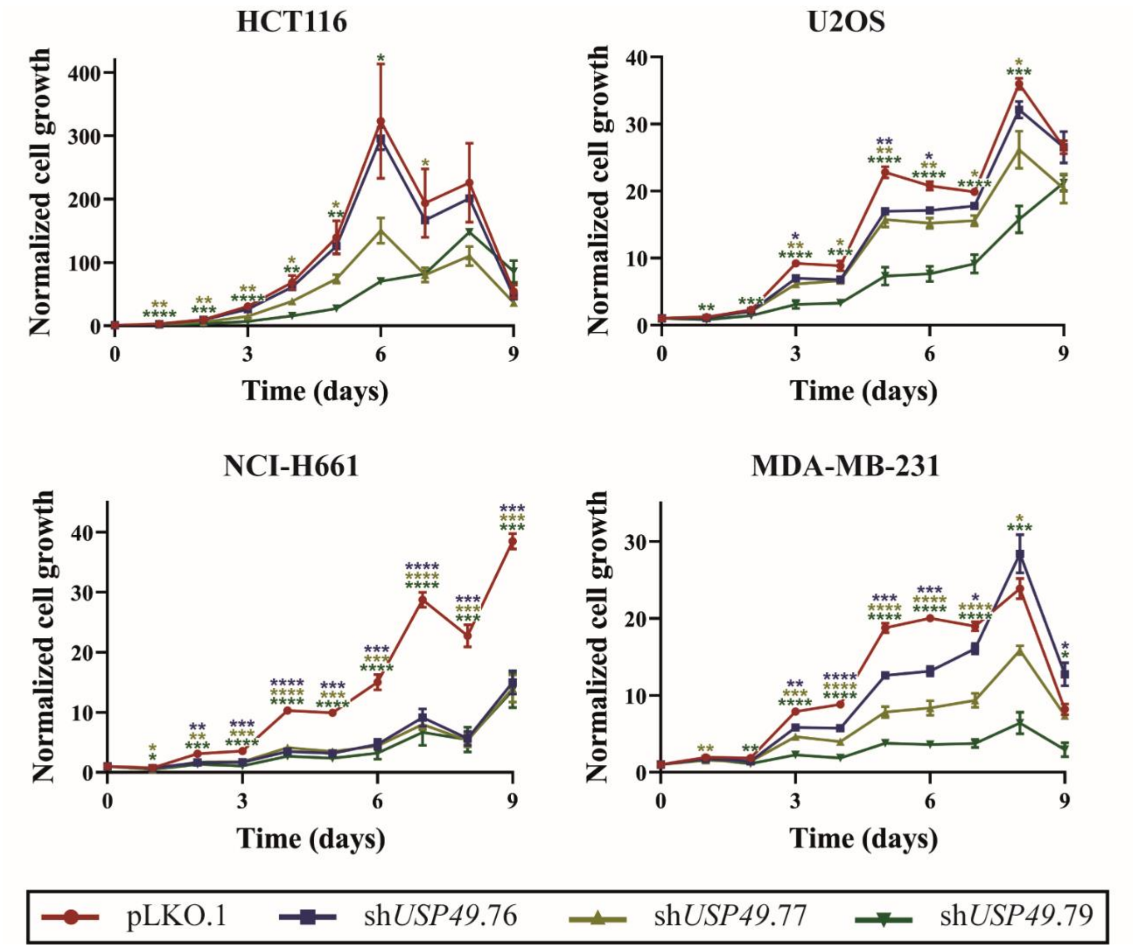

Figure S1. USP49 depletion impairs cell proliferation in a long term manner. MTT proliferation assay was performed in HCT116 (upper left), U2OS (upper right), NCI-H661 (lower left), and MDA-MB-231 (lower right) cancer cell lines every day for 10 consecutive days after being transduced with control (pLKO.1) or different *USP49*-specific shRNAs (sh*USP49*.76, sh*USP49*.77, and sh*USP49*.79). Data are presented as mean ± SEM and statistical significance was assessed by using one-way ANOVA test, setting pLKO.1 as the control column. The data corresponding only to the first 5 consecutive days in each case is also represented in **Figure 1A**, main text.

Supplementary Figure S2

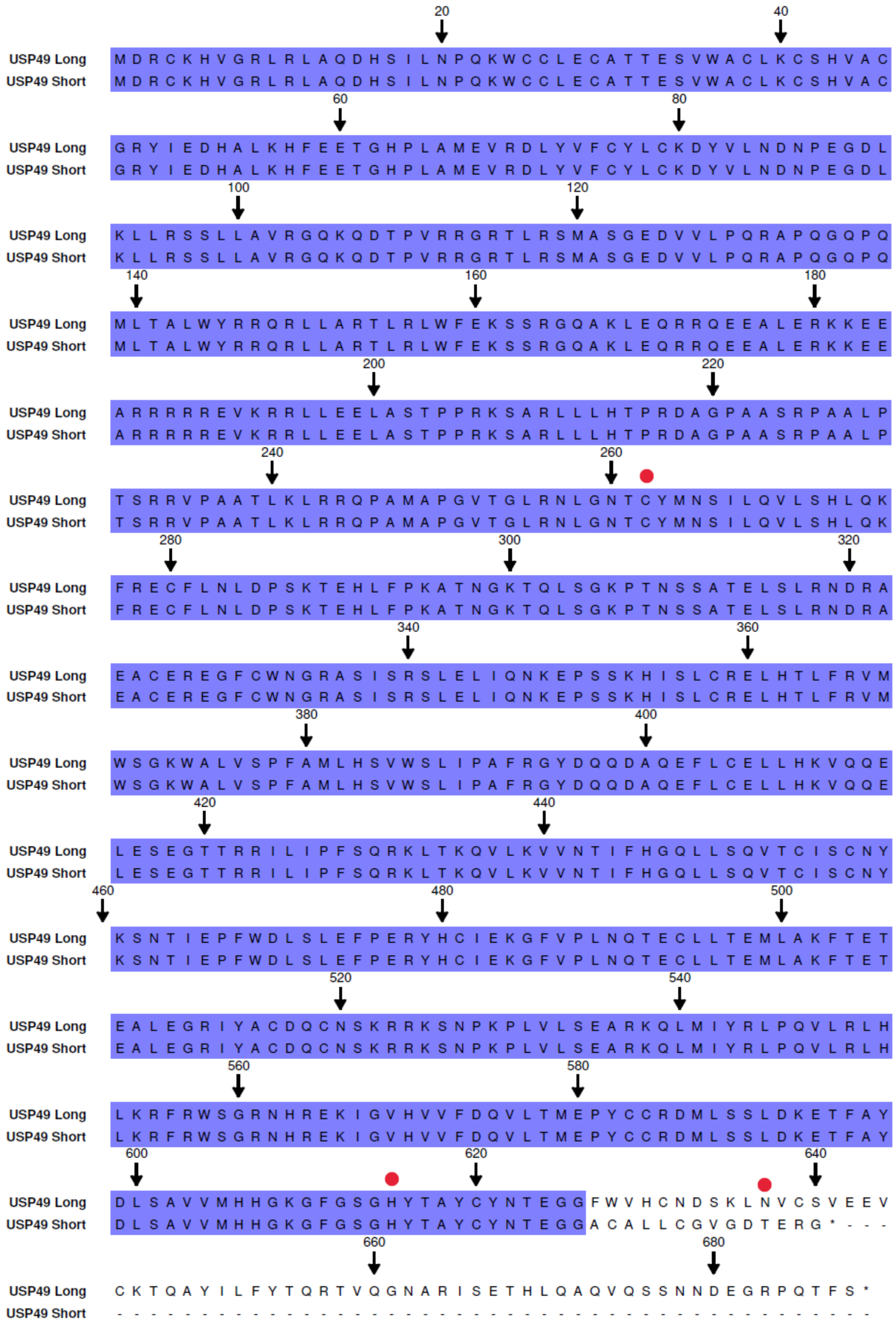

Figure S2. Amino acid sequence alignment of human USP49 long and short isoforms. The Cys, His, and Asn involved in the catalytic activity of this protease are indicated with red dots. Note that the shorter isoform lacks the Asn residue included in the catalytic triad.

Supplementary Figure S3

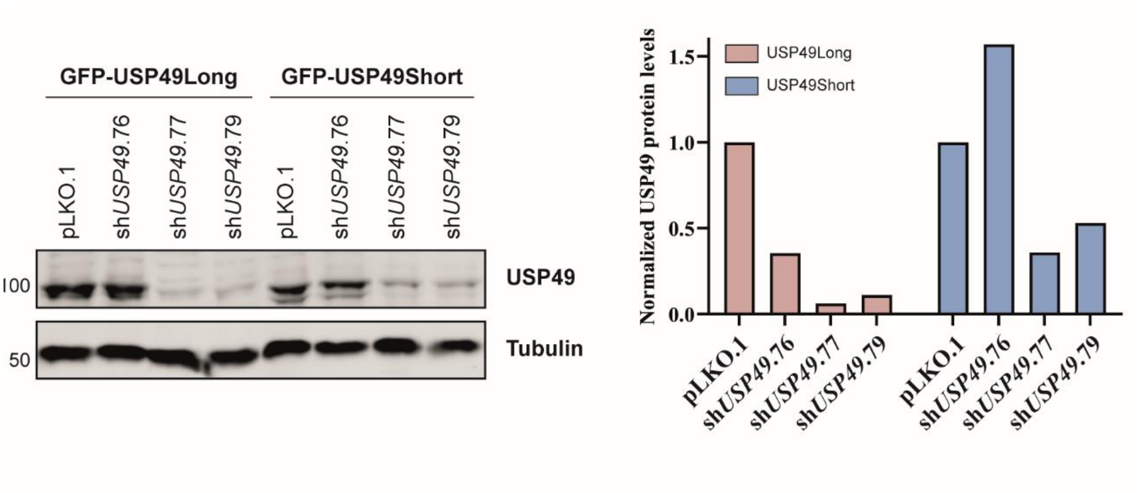

Figure S3. Exogenous USP49 protein levels decreased upon shRNA expression. Western Blot analysis and densitometry quantification of HEK-293T cells overexpressing USP49Long and USP49Short proteins in combination with pLKO.1, sh*USP49*.76, sh*USP49*.77, or sh*USP49*.79 expressing vectors.

Supplementary Figure S4

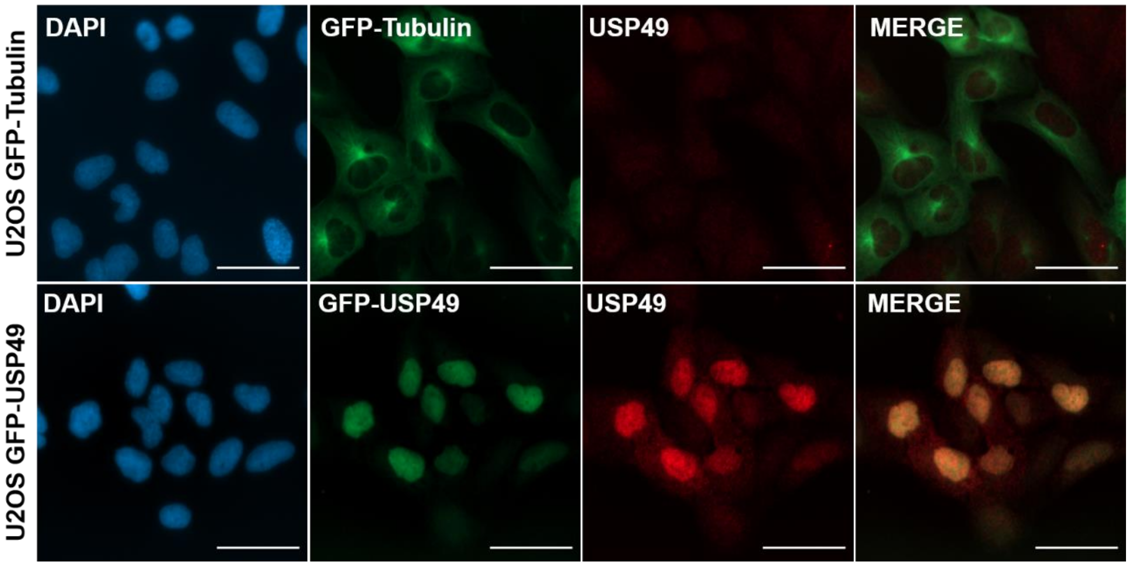

Figure S4. Subcellular localization of USP49. Immunofluorescence microscopy analysis was performed on fixed U2OS cells transduced with GFP-USP49Long or GFP-Tubulin as control. Exogenous and endogenous levels of USP49 were detected (red) using rabbit anti-USP49 antibody (HPA030255, Atlas Antibodies). DAPI was used for nuclear staining. Scale bar = 50 µm.

Supplementary Figure S5

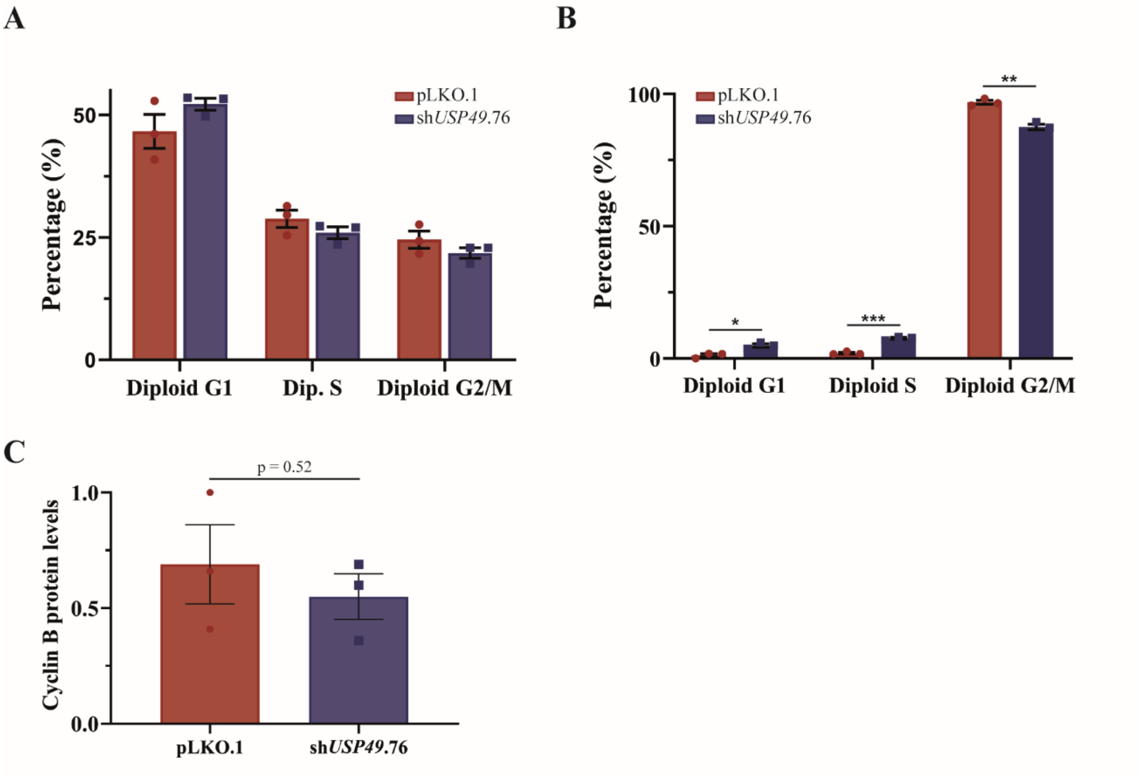

Figure S5. sh*USP49*.76 effects on HCT116 cell cycle progression. (A) HCT116 cells transduced with control or *USP49*-specific shRNA.76 were harvested for cell cycle analysis by flow cytometry in basal conditions. (B) HCT116 transduced with control or *USP49*-specific shRNA.76 were harvested for cell cycle analysis by flow cytometry after 16 h of nocodazole treatment. (C) Densitometry quantification of Cyclin B1 protein levels in HCT116 control or *USP49*-silenced using sh*USP49*.76. All represented data are the mean value ± SEM of three independent experiments Statistical significance was assessed using two-tailed Student's *t* test in all cases.

Supplementary Figure S6

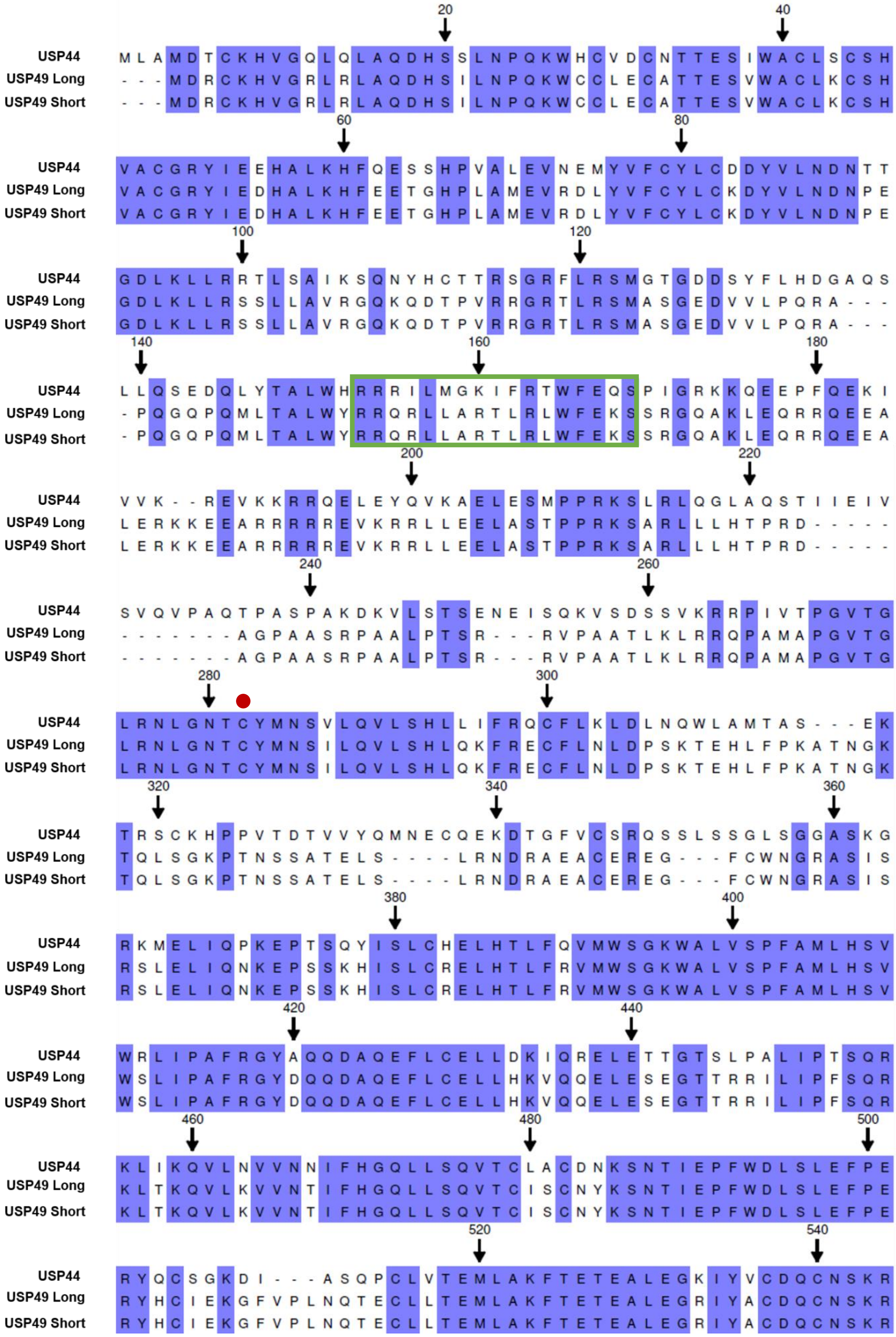

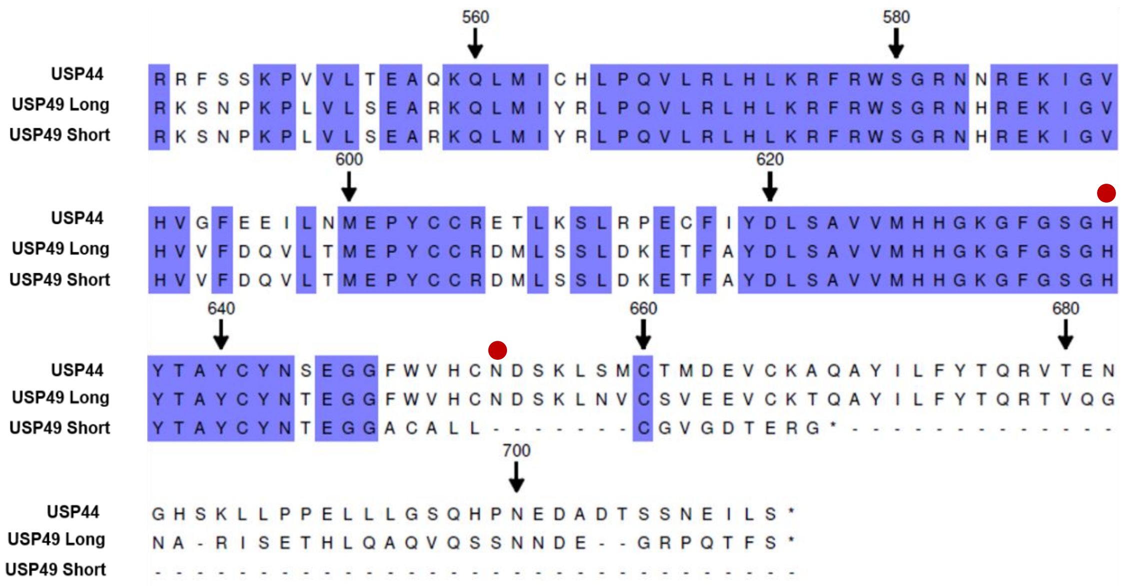

Figure S6. Amino acid sequence alignment of human USP44, and USP49 long and short isoforms. The Cys, His, and Asn involved in the catalytic activity of these proteases are indicated with red dots. The putative centrin binding domain (CBD) is indicated with a green box.

Supplementary Figure S7

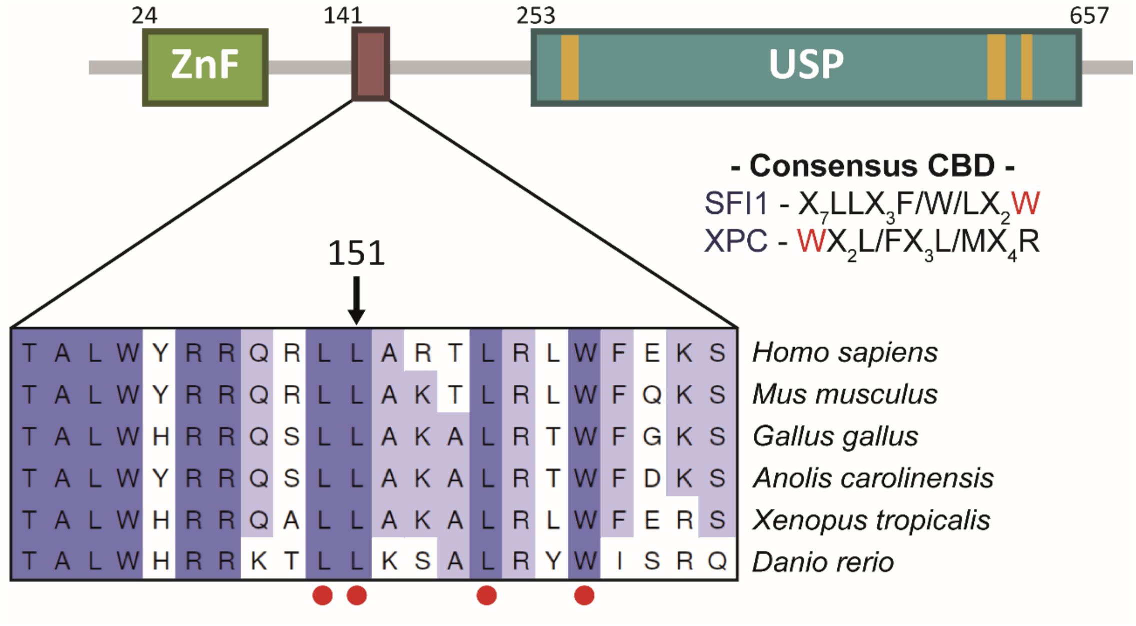

Figure S7. USP49 contains a putative centrin binding domain (CBD) highly conserved in vertebrates. (Upper) Domain map of human USP49 showing the zinc-finger ubiquitin-binding domain (ZnF), the catalytic domain (USP), and the putative centrin binding domain (CBD, marked in dark red). Positions of the residues involved in catalysis are shown in dark yellow. (Lower) Amino acid sequence alignment of the CBDs from the indicated vertebrate species. The residues marked with a red dot are the consensus residues from the XPC and SFI1 CBDs, including the essential tryptophan residue (1-3).

Supplementary Figure S8

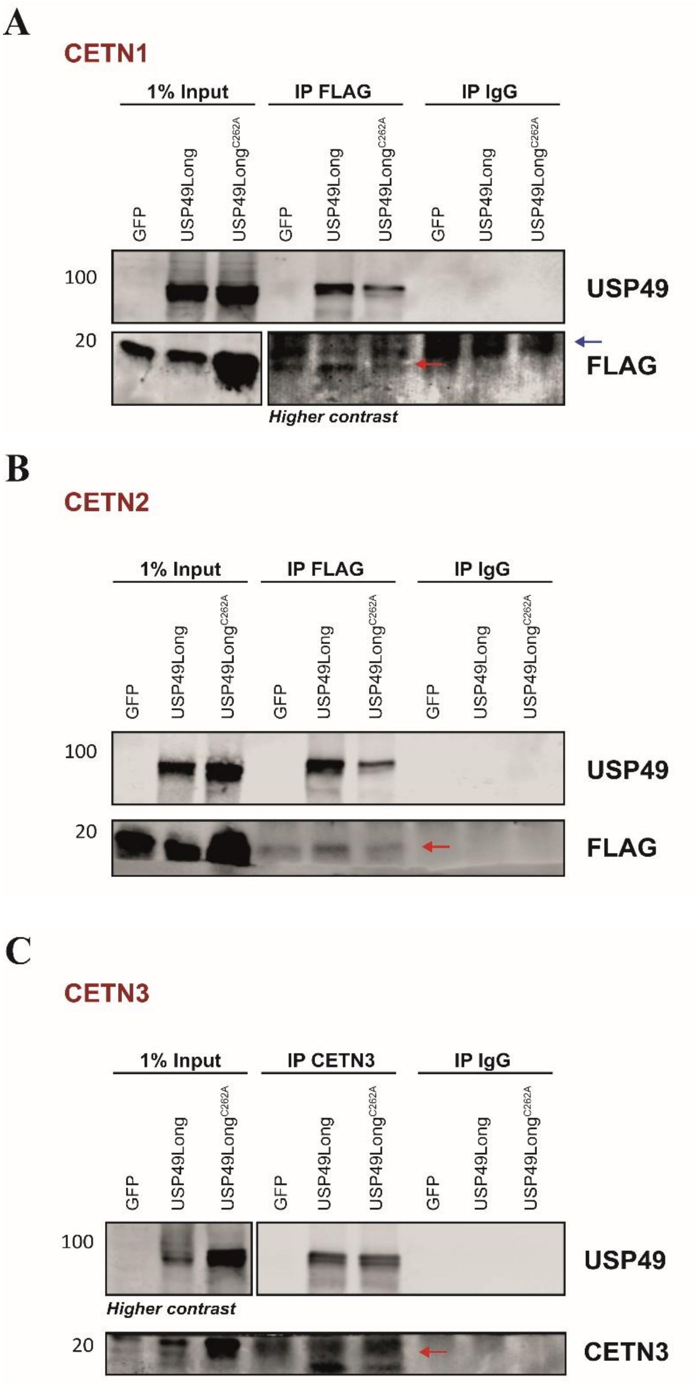

Figure S8. USP49 catalytically-dead mutant interacts with centrins. HEK-293T cells transduced with CETN1 (A), CETN2 (B), and CETN3 (C) in combination with GFP, GFP-USP49Long^{WT} or GFP-USP49Long^{C262A} were subjected to immunoprecipitation using the indicated antibodies. Precipitates were immunoblotted using anti-FLAG (A, B) or anti-CETN3 (C) antibodies. Red arrows indicate the bands corresponding to centrin, whilst the blue arrow shown in (A) indicates IgG light chain.

Supplementary References

1. Thompson JR, Ryan ZC, Salisbury JL, Kumar R. The structure of the human centrin 2-xeroderma pigmentosum group C protein complex. J Biol Chem. 2006;281:18746-18752.
2. Kilmartin JV. Sfi1p has conserved centrin-binding sites and an essential function in budding yeast spindle pole body duplication. J Cell Biol. 2003;162:1211-1221.
3. Martinez-Sanz J, Yang A, Blouquit Y, Duchambon P, Assairi L, Craescu CT. Binding of human centrin 2 to the centrosomal protein hSfi1. FEBS J. 2006;273:4504-4515.
