# Supplementary material for: USP49 deubiquitinase regulates the mitotic spindle checkpoint and prevents aneuploidy
Source: Cell Death Dis. 2023 Jan 26;14(1):60. doi: 10.1038/s41419-023-05600-x (PMC9879932; doi:10.1038/s41419-023-05600-x)
Supplement: Supplementary file 2 — Original Data File [file 41419_2023_5600_MOESM2_ESM.pdf]

Full and uncropped Western Blots for

USP49 deubiquitinase regulates the mitotic spindle checkpoint and prevents aneuploidy

Diana Campos-Iglesias[1, 2, 3], Julia M. Fraile[4], Gabriel Bretones[1], Alejandro A. Montero[1], Elena Bonzon-Kulichenko[5], Jesús Vázquez[6, 7], Carlos López-Otín[1, 2, 3] and José M. P. Freije[1, 2, 3]

[1]Departamento de Bioquímica y Biología Molecular, Instituto Universitario de Oncología del Principado de Asturias (IUOPA), Universidad de Oviedo, Oviedo, Spain.

[2]Centro de Investigación Biomédica en Red de Cáncer (CIBERONC), Madrid, Spain.

[3] Instituto de Investigación Sanitaria del Principado de Asturias (ISPA), Oviedo, Spain.

[4]Elasmogen Ltd, Liberty Building, Foresterhill Road, Aberdeen AB25 2ZP, U.K.

[5]Biochemistry Section, Regional Center for Biomedical Research (CRIB), Faculty of Environmental Sciences and Biochemistry, University of Castilla-La Mancha, Avda. Carlos III s/n, 45071 Toledo, Spain.

[6]Laboratorio de Proteómica Cardiovascular, Centro Nacional de Investigaciones Cardiovasculares (CNIC), Madrid, Spain.

[7]Centro de Investigación Biomédica en Red de Enfermedades Cardiovasculares (CIBERCV), Madrid, Spain.

Supplementary Figure S9

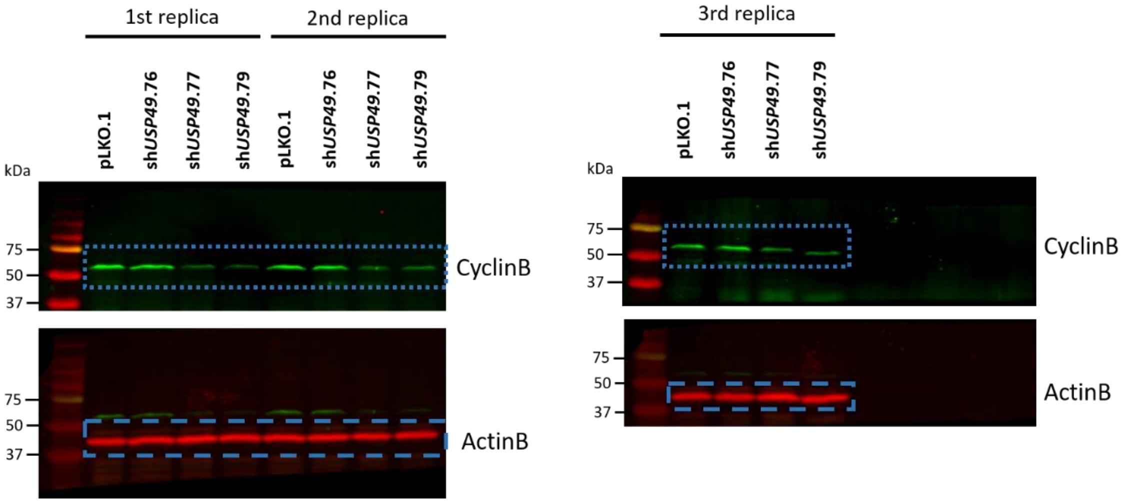

Figure S9. Raw data of Western-blots from Figure 3F, main text. The three replicates used for quantifying Cyclin B protein levels (Fig. 3F, lower panel) are shown. Lanes corresponding to the 1st replica are those included in Fig. 3F, upper panel.

Supplementary Figure S10 (see legend on page 5)

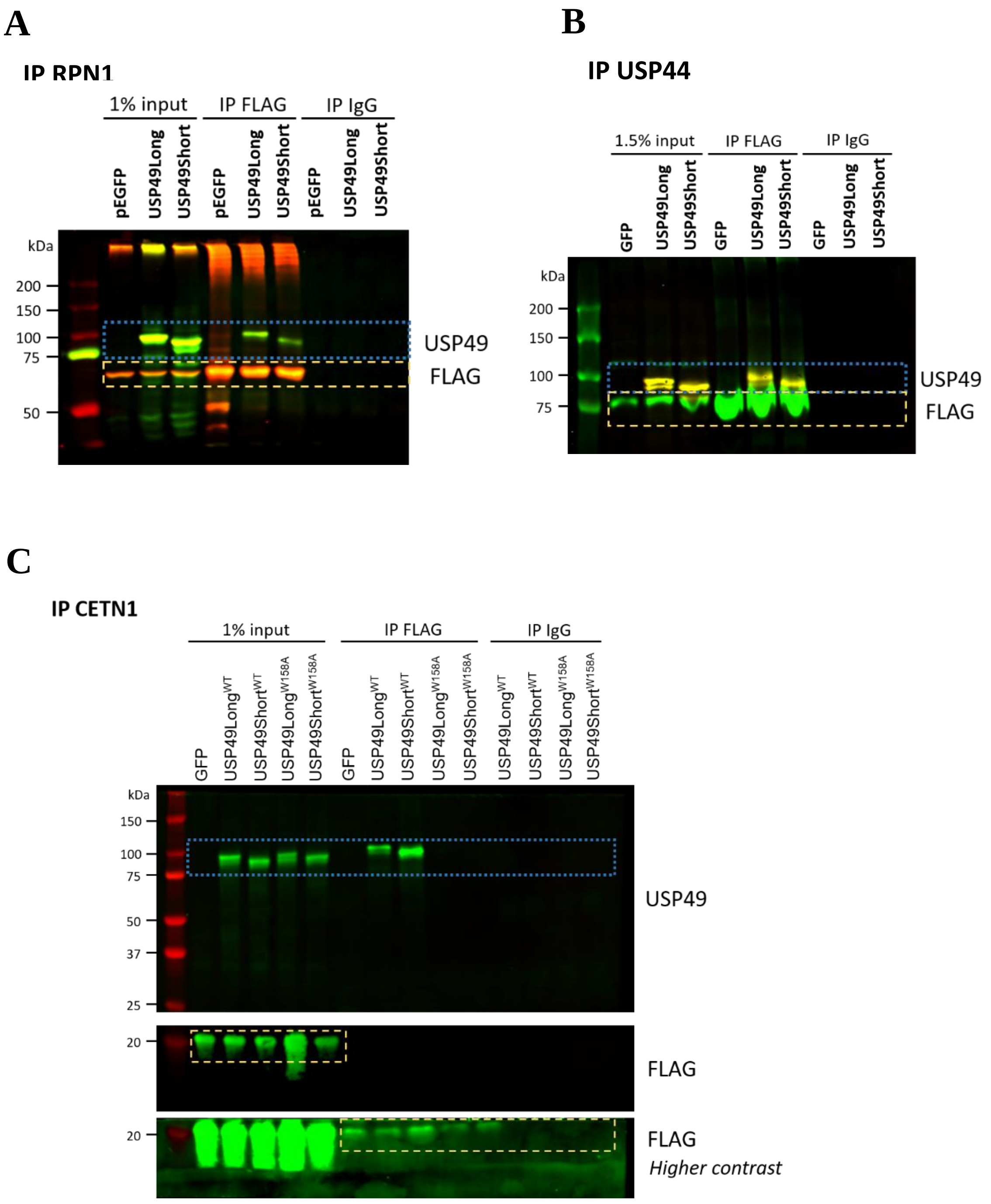

Supplementary Figure S10 (continued, see legend on page 5)

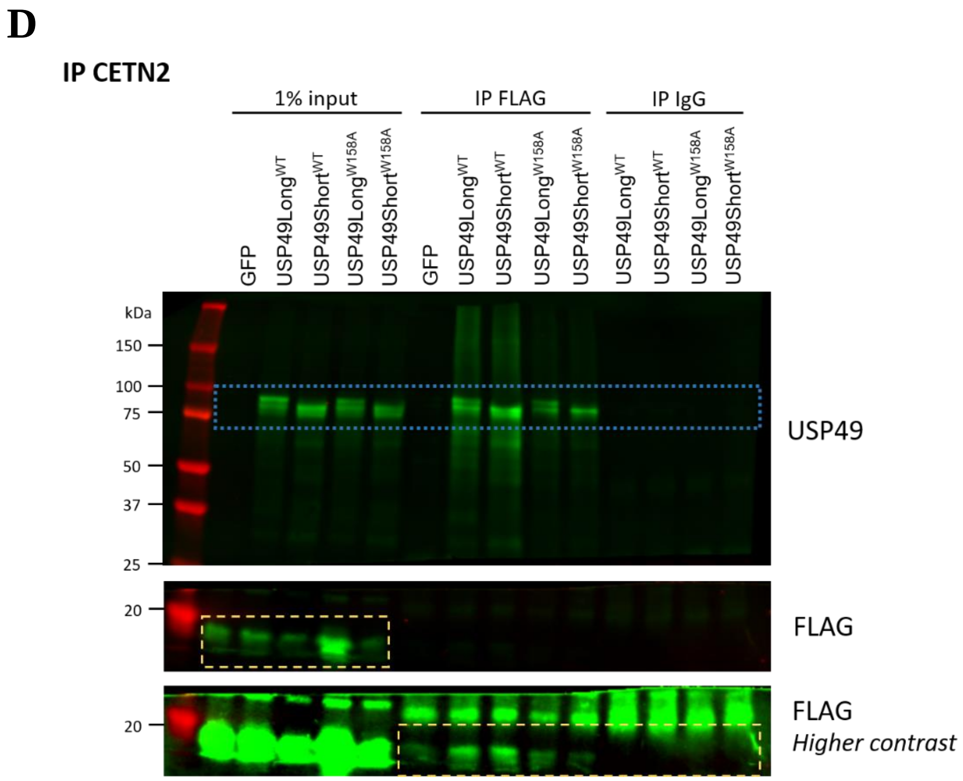

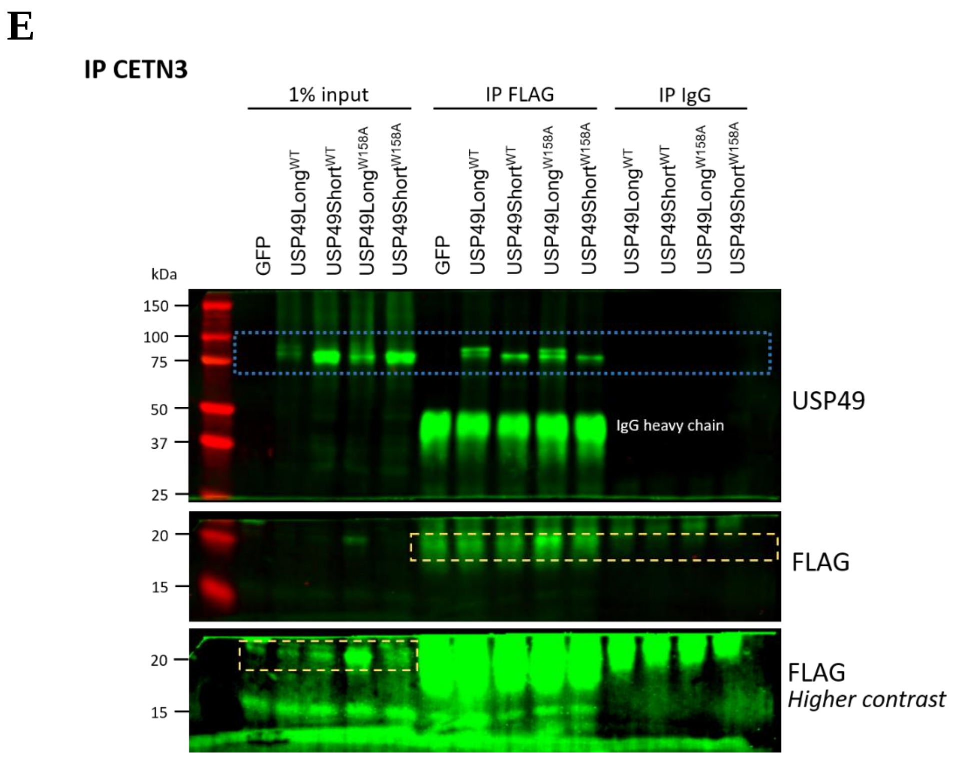

Supplementary Figure S10 (continued, see legend on page 5)

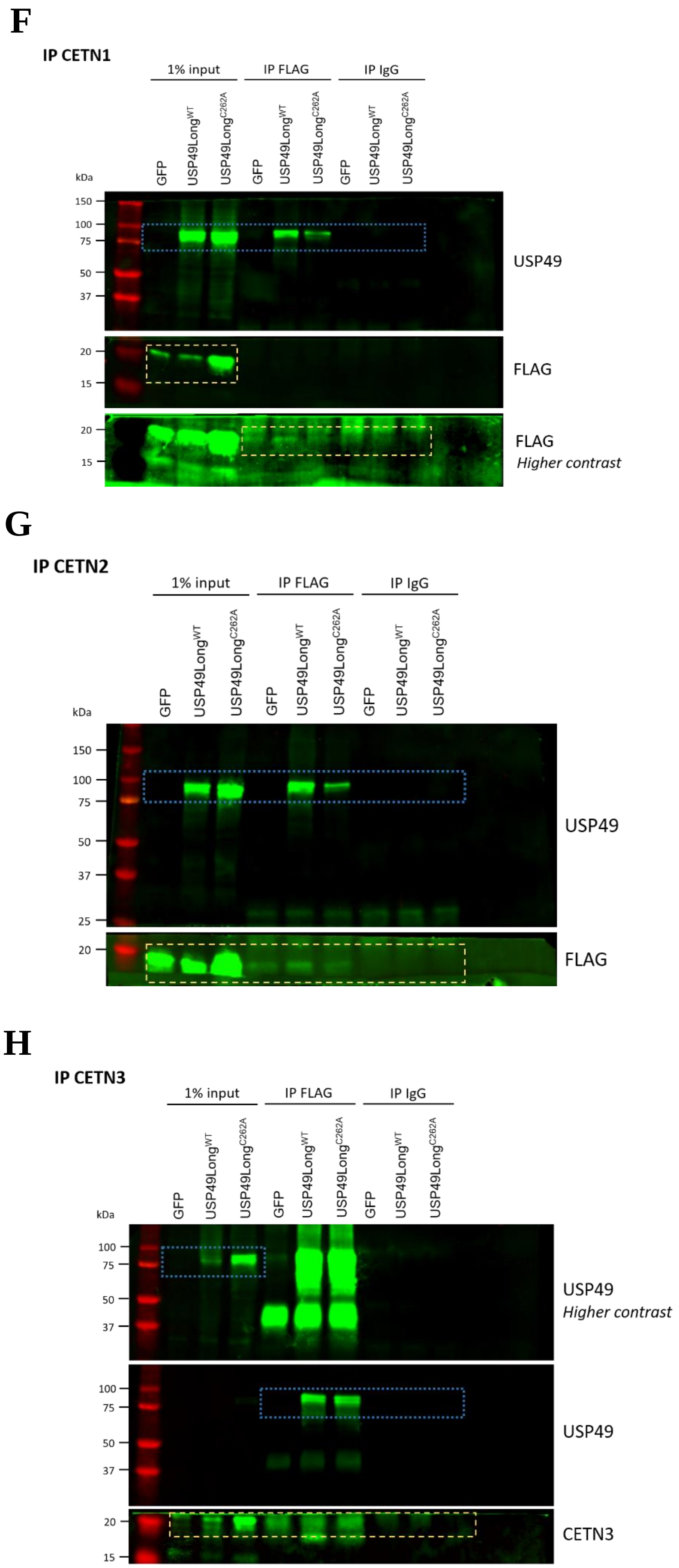

(see figure on previous pages 2, 3, and 4).

Figure S10. Raw data of Western-blots from immunoprecipitation experiments. Western-blot analysis of RPN1 (A), USP44 (B), CETN1 (C), CETN2 (D), CETN3 (E) immunoprecipitation experiments shown in Figure 4 (main text). (E, F, G) Western-blot analysis of CETN1 (F), CETN2 (G), and CETN3 (H) immunoprecipitation experiments shown in Supplementary Figure S7. Images indicated as "*Higher contrast*" have required an increment on the brightness/contrast parameters to appreciate the bands, which was performed using Image Studio software (LI-COR).
